# Association between 9-minute walk/run test and obesity among children and adolescents: evidence for criterion-referenced cut-points

**DOI:** 10.7717/peerj.8651

**Published:** 2020-02-18

**Authors:** Diego Augusto Santos Silva, Justin J. Lang, Edio L. Petroski, Júlio B. Mello, Adroaldo C.A. Gaya, Mark S. Tremblay

**Affiliations:** 1 Research Center in Kinanthropometry and Human Performance, Federal University of Santa Catarina, Florianopolis, Santa Catarina, Brazil; 2 Public Health Agency of Canada, Ottawa, ON, Canada; 3 Federal University of Rio Grande do Sul, Porto Alegre, Rio Grande do Sul, Brazil; 4 Healthy Active Living and Obesity Research Group, Children’s Hospital of Eastern Ontario Research Institute, Ottawa, ON, Canada

**Keywords:** Cardiorespiratory fitness, Physical activity, KInesiology, Obesity

## Abstract

**Background:**

Criterion-referenced cut-points for field-based aerobic fitness for children and adolescents are lacking. This study aimed to determine the associations between aerobic fitness and obesity to propose criterion-referenced cut-points for boys and girls (6–17 years).

**Methods:**

A total of 61,465 children and adolescents aged 11.2 ± 2.0 years were recruited from 27 sites (all 26 states and Federal District) across Brazil. Aerobic fitness was assessed using 9-min walk/run test reported as distance attained during the test. Body mass index was calculated from measured height and weight and was used to identify obesity.

**Results:**

The distance covered in the 9-min walk/run test (area under curve > 0.65) had satisfactory predictive ability for obesity. Sensitivity and specificity were moderate (>60%) to strong (>70%) for all age- and sex-specific cut-points. For boys, the optimal physical performance cut-points were, approximately, 1,200 m from 6 to 8 years, 1,300 m from 9 to 11 years, 1,380 m from 12 to 14 years, 1,520 m from 15 to 17 years. For girls, the best cut-points were, approximately, 1,070 m from 6 to 8 years, 1,160 m from 9 to 11 years and 1,200 m from 12 to 17 years.

**Conclusions:**

The 9-min walk/run test had satisfactory predictive ability for obesity in children and adolescents. The physical fitness cut-points proposed in the present study varied according to age and sex and could be useful and practical tools to identify low levels of physical fitness in children and adolescents in Brazil.

## Introduction

Aerobic fitness reflects the overall capacity of the cardiovascular and respiratory systems to supply oxygen during sustained physical activity (Ross et al., 2016). The benefits of adequate levels of aerobic fitness for the improvement of the cardiometabolic, cognitive and psychosocial health of children and adolescents have already been established (Lang et al., 2018). For these reasons, monitoring of aerobic fitness levels becomes an important metric for studies on population health (Ross et al., 2016). An easy and accurate way to monitor physical fitness levels is through the use of field-based tests, in which a high number of subjects are evaluated at the same time while using inexpensive equipment (Mayorga-Vega et al., 2016). The literature presents many field-based tests for the estimation of aerobic fitness in the pediatric population, and there are also many cut-points to identify the minimum amount of aerobic fitness needed for health (Boddy et al., 2012; Lang et al., 2019; Plowman & Meredith, 2013; Ruiz et al., 2016; Silva et al., 2016, 2018).

Different systematic reviews have identified that one of the field tests with less information on validity for the prediction of health indicators was the 9-min walk/run test (Mayorga-Vega et al., 2016; Lang et al., 2019; Ruiz et al., 2016; Artero et al., 2011). This test consists of running the furthest possible distance in 9 min, was developed in the mid-1970s (American Alliance for Health, Physical Education, Recreation and Dance, 1980) and was widely used by Physical Education teachers to monitor aerobic fitness in schoolchildren (American Alliance for Health, Physical Education, Recreation and Dance, 1980; Gaya & Silva, 2007). In Brazil, such test has been used since the 1990s as a tool for monitoring aerobic fitness in children and adolescents in the school environment (Gaya & Silva, 2007). Results of the aerobic fitness surveillance system in Brazil indicated that from 1990 to 2010 there was stability in the physical fitness levels of children aged 6–9 years. However, more than 80% of these children were classified with low levels of aerobic fitness (Silva, Petroski & Gaya, 2017).

A constant challenge for studies evaluating aerobic fitness levels in children and adolescents is to define the cut-points for aerobic fitness tests capable of adequately discriminating between healthy and unhealthy individuals (Ruiz et al., 2016). A meta-analysis identified seven studies that aimed to define cut-points for aerobic fitness based on cardiovascular disease risk in children and young (8–19 years). None of these studies were performed in Brazil or used the 9-min walk/run test (Ruiz et al., 2016). A review identified that only 10 studies published between 2006 and 2016 defined criterion-referenced cut-points for aerobic fitness tests in children and adolescents based on general heath indicators (Lang et al., 2019). None of these studies used the 9-min walk/run test, and only one study was conducted in Brazil. This study conducted in Brazil (Silva et al., 2016) used the modified Canadian Aerobic Fitness Test in a sample of adolescents (14–19 years) from only one city, which does not guarantee the extrapolation of the results to other regions of Brazil.

A well-studied health indicator with aerobic fitness is obesity, in which there is sufficient evidence showing that as aerobic fitness improves, body fat levels in children and adolescents decrease (Lang et al., 2018). Although there are few studies with the 9-min walk/run test and association with obesity, it is believed that, regardless of test used for aerobic fitness evaluation, the association with obesity is inverse (Lang et al., 2018). For this reason, the main purpose of this study was to define criterion-referenced cut-points, based on obesity, for children and adolescents aerobic fitness based on a sample of all regions of Brazil. This strategy will allow the aerobic fitness surveillance system in children and adolescents in Brazil to have more accurate information on cut-points for the 9-min walk/run test, since the cut-points developed for this test and used at population level are based on normative values (percent distributions), that may not provide correct classification of health risk (American Alliance for Health, Physical Education, Recreation and Dance, 1980; Gaya & Silva, 2007).

In addition, this study aims to contribute to the discussion that was recently presented in a review article (Lang et al., 2018), recommending the urgent need for standardization of cut-points for aerobic fitness in order to stimulate the creation of a system monitoring of physical fitness levels for children and adolescents. As there is no consensus on these cut-points (Boddy et al., 2012; Silva et al., 2018), the comparison between studies and estimates of prevalence of subjects with low (or high) aerobic fitness values is compromised.

Therefore, this study aimed to determine the associations between physical performance in the 9-min walk/run test and obesity to determine criterion-referenced cut-points for boys and girls (6–17 years) from all regions of Brazil.

## Materials and Methods

### Study design

This study is part of the “Brazil Sports” Project (PROESP-BR)—National Secretariat of High-Performance Sports, Ministry of Sports (Gaya & Silva, 2007). The PROESP-BR^12^ was a repeated cross-sectional surveillance study that was carried out between 1999 and 2015. It was designed to evaluate the physical fitness levels of Brazilian children and adolescents using a standardized data collection protocol across the 27 included data collection sites (all 26 states and Federal District). The aim was to recruit up to 100 participants per site, per year (from 1999 to 2016) from public and private schools in Brazil. Each site was also tasked with recruiting participants from both urban (minimum of 50% of the sample) and rural (minimum of 10% of the sample) locations. Written informed consent was obtained from parents or legal guardians. Ethics approval for this project was originally obtained from the Federal University of Rio Grande do Sul (Porto Alegre, coordinating center, Number: 2008010) (Gaya & Silva, 2007).

### Participants

Participant recruitment locations were selected across all sites using purposive, non-randomized sampling. Elementary and high schools were the primary recruitment locations for this study. Participants were considered eligible for this study if they were aged 6–17 years and maximal effort exercise was not contraindicated by a preexisting medical condition. All eligible participants were invited to participate in this study, but potential participants were able to drop out for any reason, without consequence (Gaya & Silva, 2007). Of the 85,068 participants who took part in PROESP-BR, a total of 61,465 (male = 54.3%, female = 45.7%, age 11.2 ± 2.0 years) remained in the present analysis after excluding participants without a 9-min walk/run test score (*n* = 22,033) or body mass index (BMI; *n* = 1,570) values. Children who were excluded from the present analysis due to missing data (male = 55.8%, female = 44.2%, age 11.1 ± 2.6 years) did not differ from included participants with respect to sex and age.

### Data collection procedures

Physical Education teachers from Brazilian schools were invited to participate in the study. The training of PROESP-BR was carried out online, each year, through which teachers were asked to read the project manual to learn the data collection procedures. Videos were used to demonstrate the procedure for each PROESP-BR test. Data collection procedures followed the published PROESP-BR protocol (Gaya & Silva, 2007). Data collection occurred only once for each child and occurred throughout the school year, without a month specification considering the large number of participants assessed throughout Brazil.

### Physical performance measures

Physical performance was assessed using the 9-min walk/run test following the American Alliance for Health, Physical Education, Recreation and Dance—AAHPERD procedures (American Alliance for Health, Physical Education, Recreation and Dance, 1980), that have been shown to be valid and reliable for children (Turley et al., 1994) and adolescents (Paludo et al., 2014). Students were instructed to run the furthest possible distance in 9 min. Walking during the test was allowed at any point during the test. All teachers were instructed to perform the test on a flat surface and to avoid intense sunlight during the test. The final distance covered during the test was measured in meters.

There are several equations to predict $\dot{V}{\rm O}_{2{\rm peeak}}$ using the 9-min walk/run test (15–19), but these equations were developed for specific age groups (7–12 years (Paludo et al., 2014), 8–9 years (Turley et al., 1994), 8–11 years (McCreight, 1982), 9–14 years (Baldwin, 1983), 10–14 years (Bergmann et al., 2015)), or only boys (Baldwin, 1983) or girls (McCreight, 1982). In addition, the equations use body mass, height or BMI as predictors of $\dot{V}{\rm O}_{2{\rm peeak}}$. This strategy results in multicollinearity with the outcome of this study (BMI) which limits the inference. For these reasons this research did not estimate $\dot{V}{\rm O}_{2{\rm peeak}}$ for the 9-min walk/run test.

### Obesity measures

BMI was calculated from measured height and weight. Height was measured to the nearest 0.1 cm using a stadiometer, weight was recorded to the nearest 0.1 kg using a digital weighing scale, with both reported as the average of two measures. If the measurements differed by more than 0.5 cm or 0.5 kg, a third measurement was taken and the average of the closest two measures was recorded. BMI (kg/m^2^) was subsequently derived and BMI z-scores calculated using age- and sex-specific reference data from the World Health Organization, with obesity defined as >+2 standard deviations from the mean (De Onis et al., 2007).

### Covariates

Covariates included self-reported age, geographic region of residence (North, Northeast, Midwest, Southeast and South), regular sports practice (e.g., ≥3 times/week, yes or no), and year of data collection.

### Statistical analysis

Descriptive statistics are presented as means and standard deviations, or percentages, where appropriate. Pearson correlations were calculated to quantify the relationship between physical performance in the 9-min walk/run test and obesity. Receiver-operating characteristics (ROC) were calculated to examine the discriminatory ability of physical performance in the 9-min walk/run test to predict obesity quantified by the area under the curve (AUC) (Swets, 1973). ROC curve models were adjusted by the covariates (geographic region of residence, regular sports practice, and year of data collect) (Liu & Zhou, 2013). We did one adjusted ROC curve model for the relation between the distance covered in the 9-min walk/run test and obesity. Each model was stratified by age group (6–8 years, 9–11 years, 12–14 years, 15–17 years) and sex.

Receiver-operating characteristics curves were plotted using sensitivity and specificity measures based on physical performance cut-points. A diagnostic test with AUC value equal to 1 is perfectly accurate, and a value equal to 0.5 has no discrimination power (Craig et al., 2014; Rice & Harris, 2005). AUCs values of 0.55–0.62, 0.63–0.71 and >0.71 corresponded to small, medium and large effect size (Cohen’s *d*), respectively (Rice & Harris, 2005). Sensitivity, specificity, positive predictive value, negative predictive value, positive likelihood ratio and negative likelihood ratio of physical performance were calculated at all possible cut-points to find the optimal value. The optimal value was considered the cut-point with the fewest false positives and false negatives (Habibzadeh, Habibzadeh & Yadollahie, 2016).

All analyses were performed separately for males and females. Statistical programs MedCalc 16.8.4^®^ (Ostend, Belgium) and Stata 13.0^®^ (College Station, TX, USA) were used for all analyses.

## Results

A total of 61,465 children (54.3% boys) aged 6–17 (mean age: 11.2 ± 2.0) years were retained for the present analyses. The mean BMI value was 18.5 ± 3.4 kg/m^2^. The average distance completed was 1.343 ± 320 m during the 9-min walk/run test. Table 1 shows the sample distribution according to sex.

**Table 1:** Characteristics of the sample.

	All sample (*n* = 61,465)	Boys (*n* = 33,404)	Girls (*n* = 28,061)	*p*	Effect size
	Mean (SD)	Mean (SD)	Mean (SD)		
Age (years)	11.2 (2.0)	11.2 (2.0)	11.1 (1.9)	<0.01*	0.05^†^
Weight (kg)	41.7 (12.7)	41.8 (13.2)	41.6 (11.9)	0.05	0.02^†^
Height (cm)	148.6 (13.0)	148.8 (13.7)	148.3 (12.3)	<0.01*	0.04^†^
BMI (kg/m²)	18.5 (3.4)	18.4 (3.5)	18.6 (3.4)	<0.01*	0.05^†^
9-min walk/run test (m)	1,342.5 (320.1)	1,431.5 (328.9)	1,236.6 (273.8)	<0.01*	0.64^†^
9-min walk/run test (average speed-km/h)	8.9 (2.1)	9.5 (2.2)	8.2 (1.8)	<0.01*	0.64^†^
	***n* (%)**	***n* (%)**	***n* (%)**		
Age (years)					
6–8	6,118 (10.0)	3,241 (9.7)	2,877 (10.3)	<0.01*	0.02^‡^
9–11	27,486 (44.6)	14,726 (44.1)	12,760 (45.5)		
12–14	24,997 (40.7)	13,790 (41.3)	11,207 (39.9)		
15–17	2,864 (4.7)	1,647 (4.9)	1,217 (4.3)		
Geographic region					
North	3,230 (5.3)	1,766 (5.3)	1,464 (5.2)	<0.01*	0.07^‡^
Northeast	27,149 (44.2)	2,924 (8.8)	22,62 (8.1)		
Midwest	5,186 (8.4)	3,535 (10.6)	3,027 (10.8)		
Southeast	19,338 (31.5)	11,384 (34.1)	7,954 (28.3)		
South	6,562 (10.6)	13,795 (41.2)	13,354 (47.6)		
Sports practice					
Yes	25,791 (42.0)	16,077 (48.1)	9,714 (34.6)	<0.01*	0.13^‡^
No	35,674 (58.0)	17,327 (51.9)	18,347 (65.4)		
BMI z-score (WHO)					
No obesity	56,109 (91.3)	29,877 (89.4)	26,232 (93.5)	<0.01*	0.07^‡^
Obesity	5,356 (8.7)	3,527 (10.6)	1,829 (6.5)		

**Notes:**

* *p* < 0.01.

† Cohen’s *d*.

‡ Cramer’s *V*.

SD, standard deviation; BMI, body mass index; WHO, World Health Organization.

In all ages and in both sexes there was a negative correlation between CRF and BMI (Table 2).

**Table 2:** Pearson correlation coefficient (*r*) of association between 9-min walk/run test and body mass index in boys and girls.

	Boys	Girls
	9-min walk/run test (m) × BMI (kg/m²)	9-min walk/run test (m) × BMI (kg/m²)
	*r*	*r*
6–8 years	−0.16*	−0.15*
9–11 years	−0.25*	−0.18*
12–14 years	−0.19*	−0.17*
15–17 years	−0.18*	−0.16*
6–17 years	−0.11*	−0.13*

**Notes:**

* *p* < 0.01.

BMI, body mass index; *r*, Pearson’s correlation coefficient.

**Figure 1:**
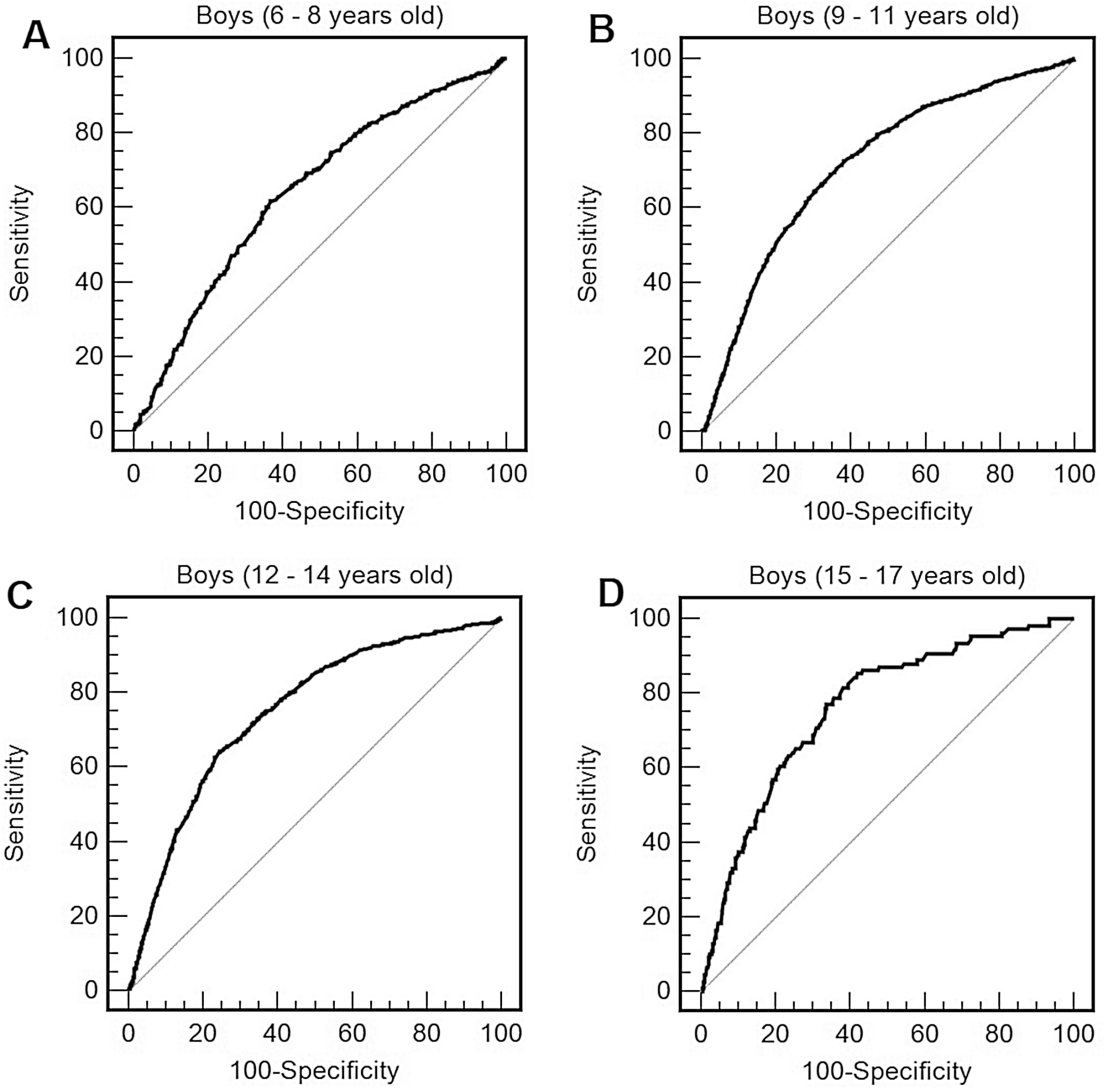
Receiver operating characteristic curve of performance in the 9 min walk/run test (distance reached in the test) to predict obesity by body mass index (BMI) in boys of 6–8 years old (A), 9–11 years old (B), 12–14 years old (C), and 15–17 years old (D).

**Figure 2:**
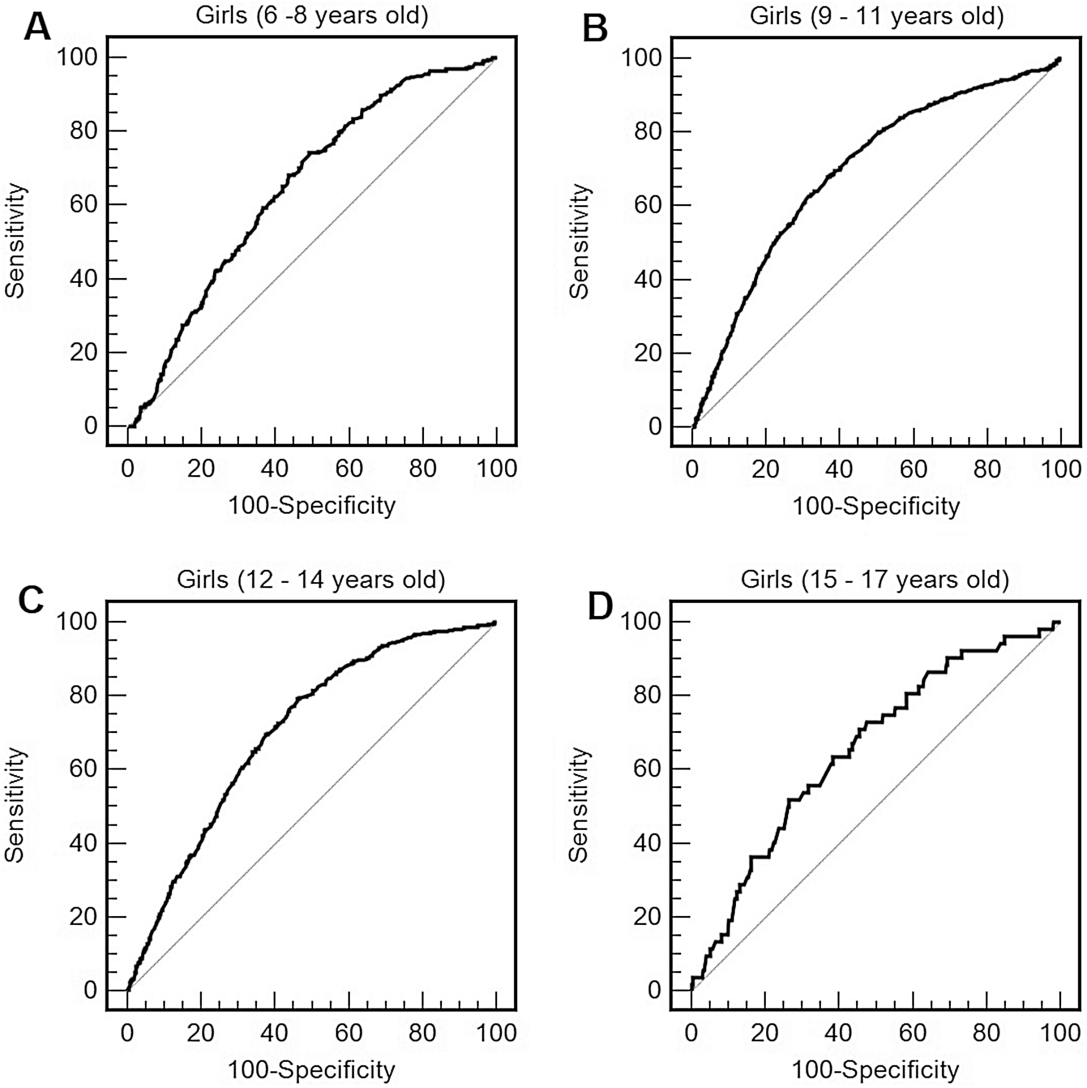
Receiver operating characteristic curve of performance in the 9 min walk/run test (distance reached in the test) to predict obesity by body mass index (BMI) in girls of 6–8 years old (A), 9–11 years old (B), 12–14 years old (C), and 15–17 years old (D).

For boys (Table 3; Fig. 1) and for girls (Table 3; Fig. 2), the distance completed during the 9-min walk/run test showed the ability to predict obesity in all ages. AUCs were above 0.65 (medium effect size) at all ages and for both sexes, with AUCs being higher for boys compared to girls. For boys, the optimal cut-points for obesity were, approximately, 1,200 m from 6 to 8 years, 1,300 m from 9 to 11 years, 1,380 m from 12 to 14 years, 1,520 m from 15 to 17 years. For girls, the best cut-points for obesity were, approximately, 1,070 m from 6 to 8 years, 1,160 m from 9 to 11 years and 1,200 m from 12 to 17 years.

**Table 3:** Diagnostic properties of 9-min walk/run test (distance reached in the test) to predict obesity in boys and girls.

	Obesity by BMI z-score								
	AUC (95% CI)	Cut-points (m)	Cut-points (average speed-km/h)	Sensitivity (%) (95% CI)	Specificity (%) (95% CI)	PPV (%)	NPV (%)	LR+	LR−
Boys									
6–8 years	0.65 [0.63–0.66]*	1,190	7.9	62.0 [58.0–67.0]	62.0 [60.0–64.0]	22.6	90.1	1.6	0.6
9–11 years	0.72 [0.71–0.73]*	1,300	8.7	71.0 [69.0–73.0]	64.0 [63.0–65.0]	21.6	93.9	2.0	0.5
12–14 years	0.75 [0.74–0.76]*	1,380	9.2	69.0 [66.0–72.0]	69.3 [68.5–70.1]	16.1	96.3	2.2	0.5
15–17 years	0.76 [0.74–0.78]*	1,520	10.1	71.0 [61.2–79.0]	69.1 [66.7–71.4]	13.9	97.1	2.3	0.4
Girls									
6–8 years	0.65 [0.63–0.67]*	1,070	7.1	62.3 [57.0–67.8]	60.3 [58.3–62.2]	15.8	93.0	1.6	0.6
9–11 years	0.70 [0.69–0.71]*	1,160	7.7	69.0 [66.0–71.6]	62.0 [61.0–62.8]	12.4	96.2	1.8	0.5
12–14 years	0.71 [0.70–0.72]*	1,200	8.0	72.0 [67.5–75.3]	60.0 [59.0–61.0]	8.3	97.6	1.8	0.5
15–17 years	0.66 [0.63–0.68]*	1,200	8.0	73.1 [59.0–84.4]	62.0 [58.8–64.4]	7.0	97.4	1.7	0.6

**Notes:**

* *p* < 0.05.

BMI, body mass index; AUC, area under the curve; 95% CI, 95% confidence interval; PPV, positive predictive value; NPV, negative predictive value; LR+, positive likelihood ratio; LR−, negative likelihood ratio.

## Discussion

The present study is consistent with other studies that reported associations between aerobic fitness and obesity in children and adolescents (Clark et al., 2015; Gonçalves, Nunes & Silva, 2017; Héroux et al., 2013). The novelty of this study is that this is the first research to find this association using the 9-min walk/run test in a sample of children and adolescents from all over Brazil and that proposed criterion-referenced cut-points for the 9-min walk/run test.

The first normative-referenced standards for the 9-min walk/run test was for the distance covered in the test and was proposed in the mid-1980s based on normative values (percentiles) of the American population (American Alliance for Health, Physical Education, Recreation and Dance, 1980). At that time, more than 12,000 students from thirteen US states were surveyed. The authors proposed values for sex and age, and values below the 50th percentile (P) were an indication that aerobic fitness needed improvement. If the participants were below P25 they should receive special attention and be encouraged to improve aerobic fitness (American Alliance for Health, Physical Education, Recreation and Dance, 1980). The limitation of this interpretation strategy is that the normative-referenced standards are not linked to health outcomes. The present study identifies criterion-referenced cut-points linked to health outcomes.

The present study found that the AUCs values of the association between obesity and physical performance and the sensitivity and specificity values of the cut-points for the distance covered during the test were moderate to strong. These results are in line with other research that used other field-based tests to estimate aerobic fitness and found that such tests are good health discriminators (Boddy et al., 2012; Lang et al., 2019; Plowman & Meredith, 2013; Ruiz et al., 2016; Silva et al., 2016, 2018). The literature has reported that $\dot{V}{\rm O}_{2{\rm peeak}}$ values below 35 and 42 mL·kg^−1^·min^−1^ for females and males aged 8–19 years, respectively, could be used to screen for individuals at risk of poor cardiovascular health (Ruiz et al., 2016). Our study did not present $\dot{V}{\rm O}_{2{\rm peeak}}$ estimates because there is not in the literature a single equation for the age group investigated. The present study adds to the literature useful information for Physical Education and health professionals, in that the estimate of the distance reached in the test is sufficient to discriminate an obese children or adolescent.

This study has some notable limitations. The first is the non-probabilistic sample. All states of Brazil had data collected in the urban and rural areas; however, due to the non-probabilistic process, the country representativeness cannot be guaranteed. Regardless, this research contributes to an attempt to standardize cut-points based on health criteria for aerobic fitness using a large sample. In addition, data from this research project are used in Brazil for physical fitness follow-up of children and adolescents (Silva, Petroski & Gaya, 2017), making the information presented relevant for the country.

The second limitation of this research is the use of BMI to classify obesity. Such measure has universal applicability, but is an estimate that has limitations when used in children and adolescents who are in the growth and development process, where high BMI does not necessarily represent increased body fat (Wells, 2014). Although BMI estimated obesity was used as the outcome of this study, it is not intended to replace the body mass and height (and BMI calculation) for obesity estimation with the 9-min walk/run test.

## Conclusions

It could be concluded that the 9-min walk/run test had satisfactory predictive ability to predict obesity in children and adolescents aged 6–17 years. Distance covered in the test was adequate measures to predict obesity in children and adolescents in Brazil. The physical performance cut-points proposed in the present study varied according to age and sex and could be useful and practical tools to identify low levels of physical performance in children and adolescents in Brazil.

## Supplemental Information

10.7717/peerj.8651/supp-1 Supplemental Information 1 Dataset.

## Additional Information and Declarations

### Data Availability

The following information was supplied regarding data availability: The raw measurements are available in the Supplemental Files.
